# μ-2,2′-(Azinodimethylene)dibenzene­sulfonato-bis­[hepta­aqua­europium(III)] bis­[2,2′-(azino­dimethylene)dibenzene­sulfonate] deca­hydrate

**DOI:** 10.1107/S1600536809006485

**Published:** 2009-02-28

**Authors:** Xi-Shi Tai

**Affiliations:** a Department of Chemistry and Chemical Engineering, Weifang University, Weifang 261061, People’s Republic of China

## Abstract

In the title compound, [Eu_2_(C_14_H_10_N_2_O_6_S_2_)(H_2_O)_14_](C_14_H_10_N_2_O_6_S_2_)_2_·10H_2_O, the complete bimetallic cation is generated by crystallographic inversion symmetry. The Eu atom adopts a distorted dodeca­hedral coordination arising from one O-bonded 2,2′-azinodibenzene­sulfonate ligand and seven water mol­ecules. In the crystal structure, the components are linked by multiple O—H⋯O and O—H⋯N hydrogen bonds.

## Related literature

For background on hybrid materials, see: Guo *et al.* (2008 ▶); Yang *et al.* (2006 ▶); Zhang *et al.* (2007 ▶).
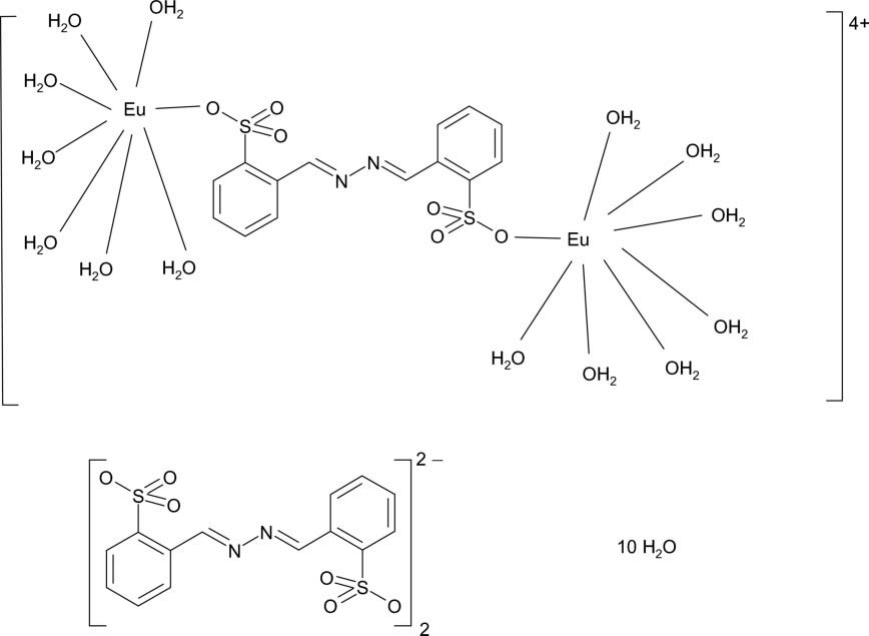


## Experimental

### 

#### Crystal data

[Eu_2_(C_14_H_10_N_2_O_6_S_2_)(H_2_O)_14_](C_14_H_10_N_2_O_6_S_2_)_2_·10H_2_O
*M* *_r_* = 1835.37
Monoclinic, 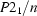

*a* = 12.5504 (14) Å
*b* = 18.611 (2) Å
*c* = 15.6699 (15) Å
β = 108.964 (2)°
*V* = 3461.5 (7) Å^3^
*Z* = 2
Mo *K*α radiation
μ = 2.08 mm^−1^
*T* = 298 K
0.30 × 0.20 × 0.12 mm

#### Data collection

Bruker SMART CCD area-detector diffractometer
Absorption correction: multi-scan (*SADABS*; Bruker, 2000 ▶) *T*_min_ = 0.574, *T*_max_ = 0.788
17265 measured reflections
6086 independent reflections
5162 reflections with *I* > 2σ(*I*)
*R*_int_ = 0.045

#### Refinement

*R*[*F* ^2^ > 2σ(*F* ^2^)] = 0.030
*wR*(*F* ^2^) = 0.083
*S* = 1.09
6086 reflections
442 parameters
H-atom parameters constrained
Δρ_max_ = 0.97 e Å^−3^
Δρ_min_ = −0.88 e Å^−3^

### 

Data collection: *SMART* (Bruker, 2000 ▶); cell refinement: *SAINT* (Bruker, 2000 ▶); data reduction: *SAINT*; program(s) used to solve structure: *SHELXS97* (Sheldrick, 2008 ▶); program(s) used to refine structure: *SHELXL97* (Sheldrick, 2008 ▶); molecular graphics: *SHELXTL* (Sheldrick, 2008 ▶); software used to prepare material for publication: *SHELXTL*.

## Supplementary Material

Crystal structure: contains datablocks global, I. DOI: 10.1107/S1600536809006485/hb2895sup1.cif

Structure factors: contains datablocks I. DOI: 10.1107/S1600536809006485/hb2895Isup2.hkl

Additional supplementary materials:  crystallographic information; 3D view; checkCIF report

## Figures and Tables

**Table 1:** Selected bond lengths (Å)

Eu1—O10	2.314 (3)
Eu1—O15	2.323 (3)
Eu1—O7	2.328 (3)
Eu1—O11	2.348 (3)
Eu1—O12	2.350 (3)
Eu1—O13	2.362 (3)
Eu1—O16	2.365 (3)
Eu1—O14	2.389 (3)

**Table 2:** Hydrogen-bond geometry (Å, °)

*D*—H⋯*A*	*D*—H	H⋯*A*	*D*⋯*A*	*D*—H⋯*A*
O10—H10*C*⋯O5^i^	0.85	1.86	2.707 (4)	173
O10—H10*D*⋯O18	0.85	1.87	2.712 (5)	174
O11—H11*C*⋯N2^ii^	0.85	2.17	3.020 (5)	175
O11—H11*D*⋯O20	0.85	1.82	2.669 (5)	174
O12—H12*C*⋯O9	0.85	1.92	2.770 (4)	179
O12—H12*D*⋯O4^iii^	0.85	1.88	2.734 (4)	179
O13—H13*C*⋯O2^iv^	0.85	1.94	2.785 (5)	172
O13—H13*D*⋯O5^i^	0.85	2.00	2.846 (5)	171
O14—H14*C*⋯O9^v^	0.85	2.11	2.939 (4)	165
O14—H14*D*⋯O17	0.85	1.83	2.660 (5)	164
O14—H14*D*⋯O15	0.85	2.40	2.838 (5)	113
O15—H15*C*⋯O17	0.85	1.96	2.811 (4)	176
O15—H15*D*⋯O21^iv^	0.85	1.82	2.671 (5)	175
O16—H16*C*⋯O6^iii^	0.85	1.94	2.779 (4)	167
O16—H16*D*⋯O19^iv^	0.85	1.96	2.798 (5)	168
O17—H17*C*⋯O1^vi^	0.85	1.91	2.749 (4)	169
O17—H17*D*⋯O3^iv^	0.85	1.90	2.736 (5)	168
O18—H18*C*⋯O3^vii^	0.85	1.99	2.787 (5)	155
O18—H18*D*⋯O20	0.85	2.26	3.056 (6)	156
O19—H19*C*⋯O6^viii^	0.85	2.18	3.029 (5)	172
O19—H19*D*⋯O8^ix^	0.85	2.07	2.916 (5)	172
O20—H20*C*⋯O2^ii^	0.85	1.93	2.784 (5)	178
O20—H20*D*⋯O19^ii^	0.85	1.95	2.797 (6)	179
O21—H21*C*⋯N3^x^	0.85	2.20	3.040 (5)	171
O21—H21*D*⋯O18^xi^	0.85	2.13	2.975 (6)	172

Symmetry codes: (i) 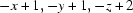; (ii) 
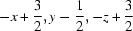
; (iii) 
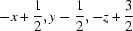
; (iv) 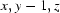; (v) 
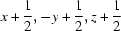
; (vi) 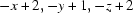; (vii) 
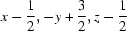
; (viii) 
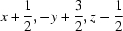
; (ix) 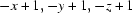; (x) 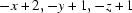; (xi) 
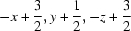
.

**Fig. 1.**
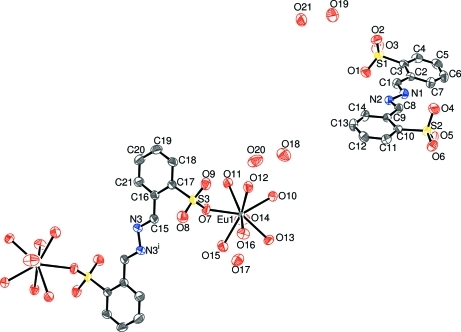
The molecular structure of (I) showing 50% displacement ellipsoids with H atoms omitted for clarity. Symmetry code: (i) 2–x, –y, 1–z.

## supplementary crystallographic information

### Comment

The design and synthesis of organic/inorganic hybrid materials have attracted intense attention in recent years owing to their potential practical applications, such as antitumor, antidiabetic, antitubercular activities, magnetism and catalysis (Zhang, *et al.*, 2007; Yang, *et al.*, 2006; Guo, *et al.*, 2008). As part of our studies in this area, we now report the synthesis and crystal structure of the title compound, (I).

The Eu(III) center is eight-coordinate with seven O donors of H_2_O and one O donor of 2-formyl-benzenesulfonate-hydrazine, and adopts triangluar dodecahedral coordination. It is interesting that one ligand coordinates to Eu(III), however, the other ligand was free as anion. The bond distances of Eu—O are in the range of 2.314 (3) Å to 2.389 (3) Å. In the crystal packing, molecules form a three-dimensional network structure linked by multiple O—H···O and O—H···N hydrogen bonds.

### Experimental

A solution of 1.0 mmol 2-formyl-benzenesulfonate-hydrazine and 1.0 mmol NaOH in 5 ml 95% ethanol was added to a solution of 0.5 mmol Eu(NO_3_)_3_.6H_2_O in 5 ml ethanol at room temperature. The mixture was refluxed for 4 h with stirring, then the resulting precipitate was filtered, washed, and dried *in vacuo* over P_4_O_10_ for 48 h. Colourless blocks of (I) were obtained by slowly evaporating from methanol at room temperature.

### Refinement

The water H atoms were located in a difference map and refined as riding in their as-found relative positions with *U*_iso_(H) = 1.2*U*_eq_(O). This has led to some short intermolecular H···H contacts and the location of these H atoms should be regarded as less certain. The other H atoms were placed geometrically (C—H = 0.93–0.97 Å) and refined as riding with *U*_iso_(H) = 1.2*U*_eq_(C) or 1.5*U*_eq_(methyl C).

### Crystal data

**Table:**

[Eu_2_(C_14_H_10_N_2_O_6_S_2_)(H_2_O)_14_](C_14_H_10_N_2_O_6_S_2_)_2_·10H_2_O	*F*(000) = 1860
*M**_r_* = 1835.37	*D*_x_ = 1.761 Mg m^−^^3^
Monoclinic, *P*2_1_/*n*	Mo *K*α radiation, λ = 0.71073 Å
Hall symbol: -P 2yn	Cell parameters from 11001 reflections
*a* = 12.5504 (14) Å	θ = 2.5–28.2°
*b* = 18.611 (2) Å	µ = 2.08 mm^−^^1^
*c* = 15.6699 (15) Å	*T* = 298 K
β = 108.964 (2)°	Block, colourless
*V* = 3461.5 (7) Å^3^	0.30 × 0.20 × 0.12 mm
*Z* = 2	

### Data collection

**Table:**

Bruker SMART CCD area-detector diffractometer	6086 independent reflections
Radiation source: fine-focus sealed tube	5162 reflections with *I* > 2σ(*I*)
graphite	*R*_int_ = 0.045
ω scans	θ_max_ = 25.0°, θ_min_ = 1.8°
Absorption correction: multi-scan (*SADABS*; Bruker, 2000)	*h* = −11→14
*T*_min_ = 0.574, *T*_max_ = 0.788	*k* = −22→22
17265 measured reflections	*l* = −18→16

### Refinement

**Table:**

Refinement on *F*^2^	Primary atom site location: structure-invariant direct methods
Least-squares matrix: full	Secondary atom site location: difference Fourier map
*R*[*F*^2^ > 2σ(*F*^2^)] = 0.030	Hydrogen site location: inferred from neighbouring sites
*wR*(*F*^2^) = 0.083	H-atom parameters constrained
*S* = 1.09	*w* = 1/[σ^2^(*F*_o_^2^) + (0.0391*P*)^2^ + 3.0558*P*] where *P* = (*F*_o_^2^ + 2*F*_c_^2^)/3
6086 reflections	(Δ/σ)_max_ = 0.002
442 parameters	Δρ_max_ = 0.97 e Å^−^^3^
0 restraints	Δρ_min_ = −0.88 e Å^−^^3^

### Special details

**Table:**

Geometry. All e.s.d.'s (except the e.s.d. in the dihedral angle between two l.s. planes) are estimated using the full covariance matrix. The cell e.s.d.'s are taken into account individually in the estimation of e.s.d.'s in distances, angles and torsion angles; correlations between e.s.d.'s in cell parameters are only used when they are defined by crystal symmetry. An approximate (isotropic) treatment of cell e.s.d.'s is used for estimating e.s.d.'s involving l.s. planes.
Refinement. Refinement of *F*^2^ against ALL reflections. The weighted *R*-factor *wR* and goodness of fit *S* are based on *F*^2^, conventional *R*-factors *R* are based on *F*, with *F* set to zero for negative *F*^2^. The threshold expression of *F*^2^ > σ(*F*^2^) is used only for calculating *R*-factors(gt) *etc*. and is not relevant to the choice of reflections for refinement. *R*-factors based on *F*^2^ are statistically about twice as large as those based on *F*, and *R*- factors based on ALL data will be even larger.

### Fractional atomic coordinates and isotropic or equivalent isotropic displacement parameters (Å^2^)

**Table:**

	*x*	*y*	*z*	*U*_iso_*/*U*_eq_	
Eu1	0.717980 (14)	0.202075 (10)	0.690603 (11)	0.02135 (8)	
N1	0.5298 (3)	0.8612 (2)	1.0179 (2)	0.0387 (9)	
N2	0.5167 (3)	0.80180 (17)	0.9592 (2)	0.0338 (8)	
N3	0.9912 (3)	0.02877 (18)	0.4694 (2)	0.0339 (8)	
O1	0.8291 (2)	0.94788 (18)	0.9989 (2)	0.0526 (8)	
O2	0.7024 (3)	1.03579 (17)	0.9072 (2)	0.0470 (8)	
O3	0.8692 (3)	1.0746 (2)	1.0261 (2)	0.0625 (10)	
O4	0.1577 (3)	0.74921 (19)	0.9297 (2)	0.0552 (9)	
O5	0.2901 (3)	0.70217 (18)	1.0666 (2)	0.0495 (9)	
O6	0.1400 (2)	0.62674 (17)	0.9743 (2)	0.0443 (8)	
O7	0.7406 (2)	0.15536 (17)	0.55973 (18)	0.0393 (7)	
O8	0.6553 (3)	0.07033 (17)	0.4401 (2)	0.0489 (8)	
O9	0.5840 (2)	0.19212 (18)	0.4299 (2)	0.0479 (8)	
O10	0.6686 (3)	0.30431 (16)	0.7529 (2)	0.0485 (8)	
H10C	0.6792	0.3056	0.8093	0.058*	
H10D	0.6389	0.3438	0.7298	0.058*	
O11	0.8052 (3)	0.29204 (16)	0.6316 (2)	0.0474 (8)	
H11C	0.8539	0.2919	0.6046	0.057*	
H11D	0.8090	0.3322	0.6583	0.057*	
O12	0.5643 (2)	0.25760 (16)	0.58281 (19)	0.0406 (7)	
H12C	0.5714	0.2377	0.5361	0.049*	
H12D	0.4955	0.2553	0.5796	0.049*	
O13	0.6910 (3)	0.17085 (18)	0.8283 (2)	0.0478 (8)	
H13C	0.6952	0.1319	0.8575	0.057*	
H13D	0.6972	0.2059	0.8644	0.057*	
O14	0.8933 (3)	0.2312 (2)	0.8027 (2)	0.0613 (10)	
H14C	0.9435	0.2603	0.8330	0.074*	
H14D	0.9074	0.1902	0.8277	0.074*	
O15	0.8140 (3)	0.09405 (18)	0.7304 (2)	0.0506 (8)	
H15C	0.8630	0.0986	0.7824	0.061*	
H15D	0.8299	0.0569	0.7055	0.061*	
O16	0.5744 (2)	0.11402 (17)	0.6513 (2)	0.0467 (8)	
H16C	0.5094	0.1246	0.6154	0.056*	
H16D	0.5862	0.0698	0.6444	0.056*	
O17	0.9705 (3)	0.1154 (2)	0.9034 (2)	0.0611 (10)	
H17C	1.0359	0.0979	0.9279	0.073*	
H17D	0.9310	0.1058	0.9368	0.073*	
O18	0.5734 (3)	0.4264 (2)	0.6670 (2)	0.0700 (11)	
H18C	0.5245	0.4252	0.6147	0.084*	
H18D	0.6387	0.4243	0.6619	0.084*	
O19	0.5824 (3)	0.9682 (2)	0.6101 (3)	0.0693 (11)	
H19C	0.6050	0.9432	0.5740	0.083*	
H19D	0.5127	0.9598	0.5994	0.083*	
O20	0.8290 (4)	0.4204 (2)	0.7115 (3)	0.0841 (13)	
H20C	0.8200	0.4562	0.6762	0.101*	
H20D	0.8562	0.4354	0.7655	0.101*	
O21	0.8525 (4)	0.9787 (2)	0.6433 (3)	0.1024 (17)	
H21C	0.9014	0.9744	0.6167	0.123*	
H21D	0.8801	0.9623	0.6966	0.123*	
S1	0.78274 (9)	1.01953 (6)	0.99621 (7)	0.0389 (3)	
S2	0.21627 (8)	0.68581 (6)	0.97605 (7)	0.0334 (2)	
S3	0.68106 (8)	0.14503 (6)	0.46277 (7)	0.0337 (2)	
C1	0.6057 (3)	0.9050 (2)	1.0162 (3)	0.0373 (10)	
H1	0.6464	0.8976	0.9768	0.045*	
C2	0.6301 (3)	0.9677 (2)	1.0768 (3)	0.0326 (9)	
C3	0.7071 (3)	1.0217 (2)	1.0740 (3)	0.0348 (10)	
C4	0.7252 (4)	1.0803 (2)	1.1317 (3)	0.0437 (11)	
H4	0.7767	1.1156	1.1294	0.052*	
C5	0.6669 (4)	1.0863 (3)	1.1927 (3)	0.0513 (13)	
H5	0.6778	1.1261	1.2305	0.062*	
C6	0.5926 (4)	1.0330 (3)	1.1972 (3)	0.0495 (12)	
H6	0.5547	1.0364	1.2391	0.059*	
C7	0.5744 (3)	0.9748 (3)	1.1399 (3)	0.0420 (11)	
H7	0.5236	0.9395	1.1436	0.050*	
C8	0.4283 (3)	0.7669 (2)	0.9572 (3)	0.0347 (9)	
H8	0.3845	0.7840	0.9906	0.042*	
C9	0.3928 (3)	0.7004 (2)	0.9042 (3)	0.0297 (9)	
C10	0.3032 (3)	0.6580 (2)	0.9118 (2)	0.0294 (9)	
C11	0.2751 (3)	0.5939 (2)	0.8646 (3)	0.0358 (10)	
H11	0.2178	0.5653	0.8717	0.043*	
C12	0.3327 (3)	0.5726 (2)	0.8068 (3)	0.0394 (10)	
H12	0.3134	0.5299	0.7748	0.047*	
C13	0.4176 (4)	0.6143 (3)	0.7967 (3)	0.0433 (11)	
H13	0.4548	0.6003	0.7568	0.052*	
C14	0.4485 (3)	0.6773 (3)	0.8459 (3)	0.0388 (10)	
H14	0.5076	0.7046	0.8396	0.047*	
C15	0.9088 (3)	0.0687 (2)	0.4710 (3)	0.0339 (9)	
H15	0.8685	0.0567	0.5094	0.041*	
C16	0.8760 (3)	0.1328 (2)	0.4139 (3)	0.0319 (9)	
C17	0.7786 (3)	0.1729 (2)	0.4086 (2)	0.0305 (9)	
C18	0.7535 (3)	0.2357 (2)	0.3577 (3)	0.0371 (10)	
H18	0.6899	0.2622	0.3554	0.045*	
C19	0.8230 (4)	0.2588 (3)	0.3106 (3)	0.0444 (11)	
H19	0.8064	0.3009	0.2770	0.053*	
C20	0.9164 (4)	0.2196 (3)	0.3134 (3)	0.0479 (12)	
H20	0.9620	0.2346	0.2804	0.058*	
C21	0.9432 (4)	0.1581 (3)	0.3648 (3)	0.0427 (11)	
H21	1.0079	0.1328	0.3669	0.051*	

### Atomic displacement parameters (Å^2^)

**Table:**

	*U*^11^	*U*^22^	*U*^33^	*U*^12^	*U*^13^	*U*^23^
Eu1	0.02425 (11)	0.02359 (13)	0.01836 (11)	−0.00072 (7)	0.00986 (8)	0.00005 (7)
N1	0.044 (2)	0.031 (2)	0.045 (2)	−0.0078 (16)	0.0205 (17)	−0.0089 (17)
N2	0.0361 (18)	0.028 (2)	0.039 (2)	−0.0034 (14)	0.0145 (15)	−0.0033 (15)
N3	0.0370 (18)	0.029 (2)	0.0354 (19)	0.0043 (15)	0.0116 (15)	0.0025 (15)
O1	0.0446 (17)	0.051 (2)	0.065 (2)	0.0102 (15)	0.0206 (16)	0.0051 (18)
O2	0.0563 (18)	0.045 (2)	0.0378 (17)	0.0002 (15)	0.0130 (14)	0.0070 (15)
O3	0.055 (2)	0.072 (3)	0.064 (2)	−0.0316 (18)	0.0231 (17)	−0.012 (2)
O4	0.0453 (18)	0.052 (2)	0.075 (2)	0.0118 (15)	0.0288 (17)	0.0145 (18)
O5	0.0439 (17)	0.074 (3)	0.0350 (17)	−0.0114 (15)	0.0189 (14)	−0.0157 (16)
O6	0.0404 (16)	0.048 (2)	0.0493 (18)	−0.0114 (14)	0.0208 (14)	0.0011 (16)
O7	0.0415 (16)	0.050 (2)	0.0281 (15)	0.0103 (14)	0.0141 (12)	−0.0004 (14)
O8	0.0535 (18)	0.040 (2)	0.057 (2)	−0.0097 (15)	0.0233 (16)	−0.0099 (16)
O9	0.0402 (17)	0.066 (2)	0.0384 (17)	0.0179 (15)	0.0134 (14)	0.0039 (16)
O10	0.074 (2)	0.040 (2)	0.0372 (17)	0.0104 (15)	0.0271 (16)	0.0008 (14)
O11	0.057 (2)	0.036 (2)	0.064 (2)	−0.0040 (14)	0.0400 (17)	−0.0004 (15)
O12	0.0343 (15)	0.050 (2)	0.0396 (16)	0.0060 (13)	0.0141 (13)	−0.0011 (15)
O13	0.077 (2)	0.0359 (19)	0.0389 (17)	−0.0045 (16)	0.0305 (16)	−0.0013 (15)
O14	0.0502 (19)	0.058 (2)	0.060 (2)	−0.0166 (17)	−0.0036 (17)	0.0076 (19)
O15	0.0548 (18)	0.057 (2)	0.0347 (16)	0.0206 (16)	0.0070 (14)	0.0017 (16)
O16	0.0367 (16)	0.0434 (19)	0.0537 (19)	−0.0046 (14)	0.0061 (14)	0.0012 (16)
O17	0.0467 (18)	0.086 (3)	0.049 (2)	0.0139 (18)	0.0135 (15)	0.020 (2)
O18	0.066 (2)	0.070 (3)	0.063 (2)	0.014 (2)	0.0059 (19)	0.001 (2)
O19	0.074 (2)	0.066 (3)	0.070 (3)	−0.001 (2)	0.025 (2)	−0.012 (2)
O20	0.137 (4)	0.057 (3)	0.064 (3)	−0.031 (3)	0.039 (3)	−0.004 (2)
O21	0.160 (4)	0.054 (3)	0.139 (4)	0.023 (3)	0.111 (4)	0.014 (3)
S1	0.0364 (5)	0.0390 (7)	0.0409 (6)	−0.0051 (5)	0.0118 (5)	0.0017 (5)
S2	0.0300 (5)	0.0397 (6)	0.0335 (5)	−0.0042 (4)	0.0143 (4)	−0.0015 (5)
S3	0.0352 (5)	0.0386 (6)	0.0288 (5)	0.0052 (4)	0.0126 (4)	−0.0012 (5)
C1	0.040 (2)	0.037 (3)	0.038 (2)	−0.0064 (19)	0.0168 (18)	−0.006 (2)
C2	0.033 (2)	0.030 (2)	0.033 (2)	0.0003 (17)	0.0079 (17)	0.0003 (18)
C3	0.035 (2)	0.030 (2)	0.036 (2)	0.0005 (17)	0.0062 (17)	−0.0008 (19)
C4	0.051 (3)	0.034 (3)	0.037 (2)	−0.004 (2)	0.001 (2)	−0.003 (2)
C5	0.062 (3)	0.042 (3)	0.040 (3)	0.011 (2)	0.004 (2)	−0.011 (2)
C6	0.047 (3)	0.061 (4)	0.040 (3)	0.009 (2)	0.013 (2)	−0.011 (2)
C7	0.037 (2)	0.051 (3)	0.039 (2)	−0.003 (2)	0.0137 (19)	−0.005 (2)
C8	0.035 (2)	0.034 (3)	0.039 (2)	−0.0050 (18)	0.0175 (18)	−0.001 (2)
C9	0.029 (2)	0.029 (2)	0.031 (2)	−0.0011 (16)	0.0105 (16)	−0.0008 (17)
C10	0.0291 (19)	0.032 (2)	0.0259 (19)	−0.0012 (16)	0.0068 (16)	0.0008 (17)
C11	0.038 (2)	0.036 (3)	0.033 (2)	−0.0048 (18)	0.0094 (17)	−0.0010 (19)
C12	0.044 (2)	0.033 (3)	0.039 (2)	−0.0006 (19)	0.0103 (19)	−0.011 (2)
C13	0.041 (2)	0.052 (3)	0.039 (2)	0.000 (2)	0.0158 (19)	−0.013 (2)
C14	0.034 (2)	0.047 (3)	0.041 (2)	−0.0034 (19)	0.0195 (19)	−0.004 (2)
C15	0.036 (2)	0.033 (2)	0.034 (2)	0.0067 (18)	0.0124 (17)	−0.0003 (19)
C16	0.035 (2)	0.031 (2)	0.029 (2)	0.0038 (17)	0.0091 (17)	−0.0015 (18)
C17	0.034 (2)	0.033 (2)	0.0228 (19)	0.0035 (17)	0.0060 (16)	−0.0008 (17)
C18	0.042 (2)	0.033 (3)	0.033 (2)	0.0085 (19)	0.0085 (19)	0.0029 (19)
C19	0.053 (3)	0.042 (3)	0.037 (2)	0.003 (2)	0.012 (2)	0.011 (2)
C20	0.050 (3)	0.053 (3)	0.045 (3)	0.000 (2)	0.022 (2)	0.010 (2)
C21	0.039 (2)	0.044 (3)	0.049 (3)	0.009 (2)	0.019 (2)	0.008 (2)

### Geometric parameters (Å, °)

**Table:**

Eu1—O10	2.314 (3)	O20—H20D	0.8500
Eu1—O15	2.323 (3)	O21—H21C	0.8499
Eu1—O7	2.328 (3)	O21—H21D	0.8500
Eu1—O11	2.348 (3)	S1—C3	1.773 (4)
Eu1—O12	2.350 (3)	S2—C10	1.784 (4)
Eu1—O13	2.362 (3)	S3—C17	1.779 (4)
Eu1—O16	2.365 (3)	C1—C2	1.473 (6)
Eu1—O14	2.389 (3)	C1—H1	0.9300
N1—C1	1.260 (5)	C2—C7	1.391 (6)
N1—N2	1.414 (5)	C2—C3	1.405 (6)
N2—C8	1.278 (5)	C3—C4	1.387 (6)
N3—C15	1.280 (5)	C4—C5	1.384 (7)
N3—N3^i^	1.406 (7)	C4—H4	0.9300
O1—S1	1.450 (3)	C5—C6	1.378 (7)
O2—S1	1.462 (3)	C5—H5	0.9300
O3—S1	1.455 (3)	C6—C7	1.377 (6)
O4—S2	1.454 (3)	C6—H6	0.9300
O5—S2	1.453 (3)	C7—H7	0.9300
O6—S2	1.452 (3)	C8—C9	1.476 (6)
O7—S3	1.472 (3)	C8—H8	0.9300
O8—S3	1.445 (3)	C9—C14	1.387 (6)
O9—S3	1.453 (3)	C9—C10	1.411 (5)
O10—H10C	0.8500	C10—C11	1.387 (6)
O10—H10D	0.8500	C11—C12	1.387 (6)
O11—H11C	0.8499	C11—H11	0.9300
O11—H11D	0.8501	C12—C13	1.368 (6)
O12—H12C	0.8499	C12—H12	0.9300
O12—H12D	0.8501	C13—C14	1.388 (6)
O13—H13C	0.8498	C13—H13	0.9300
O13—H13D	0.8500	C14—H14	0.9300
O14—H14C	0.8500	C15—C16	1.467 (6)
O14—H14D	0.8498	C15—H15	0.9300
O15—H15C	0.8500	C16—C21	1.396 (6)
O15—H15D	0.8500	C16—C17	1.411 (5)
O16—H16C	0.8500	C17—C18	1.393 (6)
O16—H16D	0.8499	C18—C19	1.381 (6)
O17—H17C	0.8501	C18—H18	0.9300
O17—H17D	0.8499	C19—C20	1.369 (7)
O18—H18C	0.8500	C19—H19	0.9300
O18—H18D	0.8500	C20—C21	1.376 (6)
O19—H19C	0.8501	C20—H20	0.9300
O19—H19D	0.8499	C21—H21	0.9300
O20—H20C	0.8501		
			
O10—Eu1—O15	141.75 (11)	Eu1—O16—H16D	123.7
O10—Eu1—O7	143.63 (11)	H16C—O16—H16D	108.1
O15—Eu1—O7	73.28 (10)	H17C—O17—H17D	108.1
O10—Eu1—O11	78.64 (12)	H18C—O18—H18D	108.9
O15—Eu1—O11	117.10 (11)	H19C—O19—H19D	108.2
O7—Eu1—O11	73.36 (11)	H20C—O20—H20D	108.4
O10—Eu1—O12	70.85 (11)	H21C—O21—H21D	108.6
O15—Eu1—O12	143.76 (11)	O1—S1—O3	112.9 (2)
O7—Eu1—O12	80.65 (10)	O1—S1—O2	111.3 (2)
O11—Eu1—O12	77.41 (11)	O3—S1—O2	111.8 (2)
O10—Eu1—O13	71.29 (11)	O1—S1—C3	107.5 (2)
O15—Eu1—O13	75.96 (11)	O3—S1—C3	105.8 (2)
O7—Eu1—O13	143.82 (12)	O2—S1—C3	107.09 (19)
O11—Eu1—O13	139.32 (12)	O6—S2—O5	112.65 (19)
O12—Eu1—O13	115.91 (11)	O6—S2—O4	112.43 (19)
O10—Eu1—O16	113.20 (11)	O5—S2—O4	112.1 (2)
O15—Eu1—O16	75.81 (11)	O6—S2—C10	106.82 (19)
O7—Eu1—O16	79.18 (11)	O5—S2—C10	107.38 (18)
O11—Eu1—O16	143.59 (11)	O4—S2—C10	104.86 (19)
O12—Eu1—O16	74.90 (11)	O8—S3—O9	113.5 (2)
O13—Eu1—O16	75.19 (11)	O8—S3—O7	112.17 (19)
O10—Eu1—O14	79.18 (13)	O9—S3—O7	111.93 (18)
O15—Eu1—O14	74.07 (12)	O8—S3—C17	107.25 (19)
O7—Eu1—O14	112.67 (11)	O9—S3—C17	106.43 (19)
O11—Eu1—O14	72.05 (12)	O7—S3—C17	104.92 (17)
O12—Eu1—O14	140.64 (12)	N1—C1—C2	119.9 (4)
O13—Eu1—O14	75.85 (12)	N1—C1—H1	120.0
O16—Eu1—O14	142.25 (11)	C2—C1—H1	120.0
O10—Eu1—H11D	62.9	C7—C2—C3	117.6 (4)
O15—Eu1—H11D	126.3	C7—C2—C1	119.2 (4)
O7—Eu1—H11D	89.4	C3—C2—C1	123.2 (4)
O11—Eu1—H11D	16.3	C4—C3—C2	120.8 (4)
O12—Eu1—H11D	77.1	C4—C3—S1	117.0 (3)
O13—Eu1—H11D	124.6	C2—C3—S1	122.2 (3)
O16—Eu1—H11D	151.1	C5—C4—C3	120.1 (5)
O14—Eu1—H11D	66.6	C5—C4—H4	120.0
O10—Eu1—H12C	88.7	C3—C4—H4	120.0
O15—Eu1—H12C	128.1	C6—C5—C4	119.7 (4)
O7—Eu1—H12C	61.8	C6—C5—H5	120.1
O11—Eu1—H12C	74.4	C4—C5—H5	120.1
O12—Eu1—H12C	18.9	C7—C6—C5	120.3 (5)
O13—Eu1—H12C	130.0	C7—C6—H6	119.8
O16—Eu1—H12C	71.7	C5—C6—H6	119.8
O14—Eu1—H12C	146.0	C6—C7—C2	121.5 (4)
H11D—Eu1—H12C	79.5	C6—C7—H7	119.2
O10—Eu1—H14D	91.1	C2—C7—H7	119.2
O15—Eu1—H14D	57.2	N2—C8—C9	122.7 (4)
O7—Eu1—H14D	110.5	N2—C8—H8	118.6
O11—Eu1—H14D	88.2	C9—C8—H8	118.6
O12—Eu1—H14D	158.7	C14—C9—C10	118.0 (4)
O13—Eu1—H14D	66.1	C14—C9—C8	120.4 (4)
O16—Eu1—H14D	124.2	C10—C9—C8	121.6 (4)
O14—Eu1—H14D	18.6	C11—C10—C9	120.5 (4)
H11D—Eu1—H14D	84.7	C11—C10—S2	117.0 (3)
H12C—Eu1—H14D	162.3	C9—C10—S2	122.4 (3)
O10—Eu1—H15C	125.8	C10—C11—C12	119.9 (4)
O15—Eu1—H15C	17.2	C10—C11—H11	120.1
O7—Eu1—H15C	87.1	C12—C11—H11	120.1
O11—Eu1—H15C	113.3	C13—C12—C11	120.3 (4)
O12—Eu1—H15C	160.7	C13—C12—H12	119.8
O13—Eu1—H15C	67.2	C11—C12—H12	119.8
O16—Eu1—H15C	88.2	C12—C13—C14	120.1 (4)
O14—Eu1—H15C	58.2	C12—C13—H13	120.0
H11D—Eu1—H15C	117.9	C14—C13—H13	120.0
H12C—Eu1—H15C	145.2	C9—C14—C13	121.2 (4)
H14D—Eu1—H15C	40.6	C9—C14—H14	119.4
C1—N1—N2	114.9 (4)	C13—C14—H14	119.4
C8—N2—N1	109.3 (4)	N3—C15—C16	121.9 (4)
C15—N3—N3^i^	112.2 (4)	N3—C15—H15	119.0
S3—O7—Eu1	142.35 (17)	C16—C15—H15	119.0
Eu1—O10—H10C	119.3	C21—C16—C17	117.1 (4)
Eu1—O10—H10D	132.2	C21—C16—C15	120.7 (4)
H10C—O10—H10D	108.5	C17—C16—C15	122.1 (4)
Eu1—O11—H11C	134.2	C18—C17—C16	120.5 (4)
Eu1—O11—H11D	113.1	C18—C17—S3	117.1 (3)
H11C—O11—H11D	108.2	C16—C17—S3	122.3 (3)
Eu1—O12—H12C	97.8	C19—C18—C17	120.1 (4)
Eu1—O12—H12D	126.9	C19—C18—H18	119.9
H12C—O12—H12D	108.4	C17—C18—H18	119.9
Eu1—O13—H13C	134.4	C20—C19—C18	120.0 (4)
Eu1—O13—H13D	114.3	C20—C19—H19	120.0
H13C—O13—H13D	108.6	C18—C19—H19	120.0
Eu1—O14—H14C	153.4	C19—C20—C21	120.4 (4)
Eu1—O14—H14D	98.0	C19—C20—H20	119.8
H14C—O14—H14D	108.0	C21—C20—H20	119.8
Eu1—O15—H15C	108.7	C20—C21—C16	121.8 (4)
Eu1—O15—H15D	139.5	C20—C21—H21	119.1
H15C—O15—H15D	108.5	C16—C21—H21	119.1
Eu1—O16—H16C	119.9		
			
C1—N1—N2—C8	−171.1 (4)	C14—C9—C10—S2	−173.9 (3)
O10—Eu1—O7—S3	−54.6 (4)	C8—C9—C10—S2	6.9 (5)
O15—Eu1—O7—S3	138.4 (3)	O6—S2—C10—C11	8.0 (4)
O11—Eu1—O7—S3	−95.8 (3)	O5—S2—C10—C11	129.1 (3)
O12—Eu1—O7—S3	−16.2 (3)	O4—S2—C10—C11	−111.5 (3)
O13—Eu1—O7—S3	105.5 (3)	O6—S2—C10—C9	−175.6 (3)
O16—Eu1—O7—S3	60.1 (3)	O5—S2—C10—C9	−54.5 (4)
O14—Eu1—O7—S3	−157.5 (3)	O4—S2—C10—C9	64.9 (4)
Eu1—O7—S3—O8	−110.2 (3)	C9—C10—C11—C12	−2.4 (6)
Eu1—O7—S3—O9	18.7 (4)	S2—C10—C11—C12	174.0 (3)
Eu1—O7—S3—C17	133.7 (3)	C10—C11—C12—C13	0.5 (6)
N2—N1—C1—C2	−177.8 (3)	C11—C12—C13—C14	1.5 (7)
N1—C1—C2—C7	4.8 (6)	C10—C9—C14—C13	−0.3 (6)
N1—C1—C2—C3	−174.8 (4)	C8—C9—C14—C13	178.8 (4)
C7—C2—C3—C4	−0.8 (6)	C12—C13—C14—C9	−1.6 (7)
C1—C2—C3—C4	178.9 (4)	N3^i^—N3—C15—C16	178.9 (4)
C7—C2—C3—S1	−179.5 (3)	N3—C15—C16—C21	−8.9 (6)
C1—C2—C3—S1	0.2 (6)	N3—C15—C16—C17	173.5 (4)
O1—S1—C3—C4	133.8 (3)	C21—C16—C17—C18	−1.4 (6)
O3—S1—C3—C4	13.0 (4)	C15—C16—C17—C18	176.4 (4)
O2—S1—C3—C4	−106.5 (3)	C21—C16—C17—S3	176.8 (3)
O1—S1—C3—C2	−47.5 (4)	C15—C16—C17—S3	−5.4 (5)
O3—S1—C3—C2	−168.3 (3)	O8—S3—C17—C18	129.5 (3)
O2—S1—C3—C2	72.2 (4)	O9—S3—C17—C18	7.8 (4)
C2—C3—C4—C5	−0.3 (6)	O7—S3—C17—C18	−111.0 (3)
S1—C3—C4—C5	178.5 (3)	O8—S3—C17—C16	−48.7 (4)
C3—C4—C5—C6	1.4 (7)	O9—S3—C17—C16	−170.5 (3)
C4—C5—C6—C7	−1.5 (7)	O7—S3—C17—C16	70.7 (4)
C5—C6—C7—C2	0.4 (7)	C16—C17—C18—C19	1.1 (6)
C3—C2—C7—C6	0.7 (6)	S3—C17—C18—C19	−177.2 (3)
C1—C2—C7—C6	−178.9 (4)	C17—C18—C19—C20	0.4 (7)
N1—N2—C8—C9	−178.1 (4)	C18—C19—C20—C21	−1.6 (7)
N2—C8—C9—C14	−7.1 (6)	C19—C20—C21—C16	1.4 (7)
N2—C8—C9—C10	172.0 (4)	C17—C16—C21—C20	0.2 (6)
C14—C9—C10—C11	2.3 (6)	C15—C16—C21—C20	−177.6 (4)
C8—C9—C10—C11	−176.8 (4)		

Symmetry codes: (i) −*x*+2, −*y*, −*z*+1.

### Hydrogen-bond geometry (Å, °)

**Table:**

*D*—H···*A*	*D*—H	H···*A*	*D*···*A*	*D*—H···*A*
O10—H10C···O5^ii^	0.85	1.86	2.707 (4)	173
O10—H10D···O18	0.85	1.87	2.712 (5)	174
O11—H11C···N2^iii^	0.85	2.17	3.020 (5)	175
O11—H11D···O20	0.85	1.82	2.669 (5)	174
O12—H12C···O9	0.85	1.92	2.770 (4)	179
O12—H12D···O4^iv^	0.85	1.88	2.734 (4)	179
O13—H13C···O2^v^	0.85	1.94	2.785 (5)	172
O13—H13D···O5^ii^	0.85	2.00	2.846 (5)	171
O14—H14C···O9^vi^	0.85	2.11	2.939 (4)	165
O14—H14D···O17	0.85	1.83	2.660 (5)	164
O14—H14D···O15	0.85	2.40	2.838 (5)	113
O15—H15C···O17	0.85	1.96	2.811 (4)	176
O15—H15D···O21^v^	0.85	1.82	2.671 (5)	175
O16—H16C···O6^iv^	0.85	1.94	2.779 (4)	167
O16—H16D···O19^v^	0.85	1.96	2.798 (5)	168
O17—H17C···O1^vii^	0.85	1.91	2.749 (4)	169
O17—H17D···O3^v^	0.85	1.90	2.736 (5)	168
O18—H18C···O3^viii^	0.85	1.99	2.787 (5)	155
O18—H18D···O20	0.85	2.26	3.056 (6)	156
O19—H19C···O6^ix^	0.85	2.18	3.029 (5)	172
O19—H19D···O8^x^	0.85	2.07	2.916 (5)	172
O20—H20C···O2^iii^	0.85	1.93	2.784 (5)	178
O20—H20D···O19^iii^	0.85	1.95	2.797 (6)	179
O21—H21C···N3^xi^	0.85	2.20	3.040 (5)	171
O21—H21D···O18^xii^	0.85	2.13	2.975 (6)	172

Symmetry codes: (ii) −*x*+1, −*y*+1, −*z*+2; (iii) −*x*+3/2, *y*−1/2, −*z*+3/2; (iv) −*x*+1/2, *y*−1/2, −*z*+3/2; (v) *x*, *y*−1, *z*; (vi) *x*+1/2, −*y*+1/2, *z*+1/2; (vii) −*x*+2, −*y*+1, −*z*+2; (viii) *x*−1/2, −*y*+3/2, *z*−1/2; (ix) *x*+1/2, −*y*+3/2, *z*−1/2; (x) −*x*+1, −*y*+1, −*z*+1; (xi) −*x*+2, −*y*+1, −*z*+1; (xii) −*x*+3/2, *y*+1/2, −*z*+3/2.
